# Treatment Changes in General Practice Patients With Chronic Mental Disorders Following a Psychiatric–Psychosomatic Consultation

**DOI:** 10.1177/2333392818758523

**Published:** 2018-03-15

**Authors:** Michael Linden, Beate Muschalla, Nils Noack, Christoph Heintze, Susanne Doepfmer

**Affiliations:** 1 Psychosomatic Rehabilitation Research Group, Charité University Medicine, Berlin, Germany; 2 Institute of General Medicine and Family Medicine, Charité University Medicine, Berlin, Germany

**Keywords:** primary care, quality assurance, chronic mental disorders, rehabilitation, general practitioner

## Abstract

**Objective:**

To determine whether a psychiatric–psychosomatic consultation can identify unmet treatment needs and improve treatment of patients with mental disorders in general practice.

**Methods:**

In 40 primary care practices, 307 consecutive primary patients who met criteria for chronic mental disorders were assessed by a psychiatric–psychosomatic consultant. After random assignment, general practitioners (GPs) were informed for half of the patients about the results of the assessment and received recommendations on how to improve treatment. Changes in treatment and patient status were reevaluated after 6 months.

**Results:**

Patients were mostly having depression, adjustment, or anxiety disorders, with 28.8% on sick leave. Contact with their respective GPs was longer than a year in 77.2% of cases. Patients had already received pharmacotherapy (60.9%), psychotherapeutic counseling by GPs themselves (27.7%), psychotherapy by specialists (73.9%), psychiatric outpatient care (57%), inpatient psychiatric treatment (12.1%), inpatient psychosomatic rehabilitation (ie, specialized behavioral medicine facilities for patients with work problems; 41.4%), and a broad spectrum of other diagnostic and therapeutic measures. Newly recommended interventions included leisure activities (42%), a new specialist psychotherapy (37.5%), or inpatient psychosomatic treatment (15.3%). Most recommendations were agreed upon by the GP. Nevertheless, there was only a limited increase in therapeutic actions 6 months later, and no statistically significant improvement in the status of patients.

**Conclusion:**

General practitioners undertake a broad spectrum of therapeutic interventions in patients with chronic mental disorders. According to our results, additional psychiatric–psychosomatic consultations can intensify treatment but does not significantly change the general course of chronic mental disorders.

## Introduction

Patients with mental disorders are treated in large numbers by general practitioners (GPs).^1-5^ General practitioners are experienced in caring for these patients, can easily be contacted, can meet with patients regularly for many years, have access to a large spectrum of treatment options, and can refer patients to specialists.^3^ Nevertheless, there is a long-standing discussion on the quality of care in general practice.^6-8^ But, final conclusions are still open for debate. Many mental disorders are by their very nature chronic, which implies that they are treatment-resistant.^9^ Therefore, it is difficult to say which treatments may be helpful or needed and at which point they should be employed in the course of the illness. There is a lack of valid guidelines for treatment across the patient’s life span.^10,11^ There are also psychological problems in the treatment of chronic illnesses, as physicians and patients may resign themselves to the state of affairs and no longer consider new options.^12-14^ This can result in undertreatment or prolonged treatments that are no longer necessary or helpful.

A method to evaluate care is to have a specialist assess individual patients, collect detailed information on the type and course of illness, and examine the success of past and present treatments.^15-17^ The specialist then can give a second opinion on the case, recommendations on what might additionally be done, and a clinical perspective on unmet or unnecessary treatments.

Given this background, we sent a psychiatric–psychosomatic specialist into the practices of GPs; they personally examined patients with chronic mental disorders, interviewed the GPs, and gathered details on the illness and treatment history. This procedure allows the specialist to make an informed judgment on the appropriateness of care and potential oversights. Next, the consultant informed the GPs about the results of the assessment for half of the cases, along with recommendations to improve treatment. Six months later, we investigated what had changed in the treatment and status of each patient. This allowed us to identify effects with respect to procedures and outcomes.

## Methods

### Physicians and Patients

Recruitment of physicians started with the register of GPs in Berlin and Brandenburg, Germany. We contacted 300 GPs and 40 were willing to participate. These physicians are “prototypically representative” for their profession in the sense of good clinical practice, as they were open to cooperate in research, which can be taken as a sign of excellence. Female physicians made up 59% of the sample, and the average age was 52.3 years (standard deviation [SD] = 7.5, range: 38-71). They had 1 to 22 years (mean = 12.6, SD = 6.2) of GP practice experience and cared, on average, for 1115 (range: 350-2300) patients per 3 months, which is the time period for reimbursement in health insurance.

A research assistant contacted 2790 consecutive patients in the waiting rooms, of which 1451 were 18 to 60 years old. Of these, 569 fulfilled the inclusion criteria for the study:

- a score of “0” or “1” in at least 1 item, or a score of “2” in at least 3 items of the World Health Organization (WHO)-5 self-rating of mental problems^18^;
- an disorder lasting longer than 6 months; and
- an average score of at least “4,” or at least 1 score of “5,” on an index for participation impairment in different life domains (IMET^19^).

These are patients with persisting mental disorders and restrictions in participation (ie, problems in the conduct of life). We did not include patients with acute or remitted illnesses. Of the eligible patients, 307 agreed to participate in an in-depth assessment and were examined by the consultant. After 6 months, we contacted physicians and patients again. Full data (including follow-up) were available from 248 patients.

### Assessment of Patients and Treatment

Patients were examined by a research physician who otherwise works full time as a consultant for psychiatric–psychosomatic medicine in an inpatient department for behavioral medicine. A special appointment was made so that there was enough time for a detailed assessment. This included (1) the medical history; (2) sociodemographic data, (3) the Mini International Neuropsychiatric Interview (MINI)^20^; (4) the Symptom Checklist (SCL-90-R), a self-rating of current psychosomatic symptoms^21^; (5) the assessment of capacity impairments and participation restrictions in patients with psychological problems, in reference to the International Classification of Functioning, Disability, and Health^22^; (6) the work anxiety interview (Arbeitsangstinterview)^23^; (7) the Index for the Measurement of Impairment in participation (IMET), a self-rating and observer rating of participation restrictions across all areas of life^16^; (8) a survey of acute and chronic somatic illnesses (the Burvill scale),^24^ a systematic compilation, and evaluation of previous and present medications; and (9) the pretreatment and next-treatment checklist (PN checklist).^25^

The core instrument was the 76-item TOPPP checklist^3^ (treatment options for persisting psychological problems). It lists all interventions which can be done in the treatment of patients with mental disorders, covering (1) diagnostic measures by the GP (“GP diagnosis”: 4 items on somatic, psychological, test, and instrument-based assessments), (2) treatments by the GP (“GP treatment”: 12 items on medication, patient education and counselling, or advice to families), (3) quality assurance and treatment structuring by the GP (“quality assurance”: 5 items on treatment monitoring, arrangements with third parties, home visits, written consent), (4) referral to specialists (“referral”: 20 items, including specialist psychotherapy, physiotherapy, special patient education, and training groups), (5) contact with institutions (“institutions”: 7 items, such as contact to health or pension insurance, child, and youth welfare), (6) certificates (“certificates”: 5 items, such as certificates of incapacity, application for invalidity pension), (7) work-related interventions (“work”: 12 items on assessment of work related capacities, demands and problems, contact with the employer, and initiation of occupational training), and (8) social support (“social support”: 10 items, including leisure activities, self-help and contact groups, home care, and supported living). The consultant filled in the TOPPP checklist after interviewing the patient and the GP. All answers refer to the present phase of the mental disorder. Past treatments are those which already have been done (eg, inpatient treatment) and present are those which are ongoing (eg, specialist psychotherapy). The results allowed us to describe the GPs’ treatment repertoire, treatment history, and possible future treatments for individual patients.

### Intervention

A senior psychiatrist (M.L.) reviewed the case reports and gave treatment recommendations, as a second opinion. The 307 patients were then assigned to an intervention group (IG; n = 153) or a control group (CG; n = 154), depending on the intake registration number, which was given to the consultant after the examination of the patient. In the CG, no information or recommendations were given to the GP. In the IG after the patient visit, the consultant discussed their findings with the GP and gave recommendations on how to proceed. There was no direct intervention in the treatment or prescription of what must be done. The GPs were free to treat the patient, independently of any recommendation, according to their personal judgment and discretion.

### Follow-Up

After 6 months, a follow-up was carried out. All 307 patients were first contacted by post and again by phone. There was in total a dropout rate of 19.2%; we analyzed data from 121 patients in the CG and 127 in the IG. Patients were asked to fill in the SCL-90,^21^ WHO-5,^18^ Burvill scale,^24^ IMET,^19^ and a questionnaire on their work status. For all patients, we interviewed GPs about the interventions they had implemented during the previous 6 months. For patients in the IG, we asked GPs to determine whether the recommendations had been useful or not using a 5-point Likert scale (rating: very useful, somewhat useful, undecided, not particularly useful, and not useful at all).

### Data Analysis and Ethical Approval

Descriptive data are reported for patient characteristics and frequency of interventions. We used χ^2^ tests to compare frequencies and groups and performed analysis of variances (ANOVAs) to test for changes over time between groups. Group sizes allowed us to test for α < .05 with an effect size of 0.2.

Approval for the study was given by the ethics committee of the Charité University of Medicine Berlin (EA4/097/09), and the internal review board of the Federal German Pension Fund (8011-106-31/31.51.6). All patients gave their written informed consent.

## Results

### Patient Characteristics

Of 307 patients, the mean age was 43.2 (SD = 10.8) years, 70.4% were female. A considerable proportion was on sick leave (28.8%) and had a disability certificate (21.5%). The most frequent diagnoses according to the standardized MINI were depression (47%), adjustment disorder (32%), agoraphobia (25%), panic disorder (19%), and alcohol abuse (8%). Some had a workplace phobia (10%).^26^ Of the patients, 77.2% had been with the same GP for more than a year, and 54.7% had more than 3 GP visits during the previous 6 months.

### Past or Present Interventions

With respect to diagnostic and therapeutic measures as performed by the GPs themselves, 83.7% of 307 cases already had a comprehensive somatic workup and 23.5% had a structured psychopathological diagnostic workup. The majority of patients had started medication (60.9%), and some had stopped medication (15.6%). Special psychotherapeutic counseling by the GP had already been given to 27.7% of patients and others (15%) had patient education using bibliotherapy (ie, some type of written materials).^27^ With respect to employment, 11.1% and 10.4% of patients were assessed for work capacity and individual work demands, respectively.

Regarding diagnostic and therapeutic measures in collaborating with other experts, 73.9% of patients had already been in specialist psychotherapy, 57.0% had seen a psychiatrist, 56.4% had physiotherapy, 23.1% relaxation/stress management courses, and 17.6% had contact with self-help groups. Additionally, 12.1% had been in psychiatric inpatient treatment, and 41.4% had been in psychosomatic inpatient treatment.^28^

Regarding interventions in cooperation with health insurance or similar institutions, there had been contacts with case managers of state work agencies in 22.8% of cases and with health insurance in 11.1%. In 5.5%, social psychiatric services were contacted. Measures of social support were primarily to propose leisure activities in 11.7%, credit counseling in 5.5%, educational counseling in 1.6%, or domestic help in 1.0%.

However, there were no therapeutic group treatments or self-help groups provided in the GP practice. There were no special laboratory tests, such as medication serum levels, and GPs did not intervene with treatments initiated by other physicians.

### Consultant Recommendations

For 89.6% of all 307 patients, at least 1 recommendation was given, with a range of 0 to 15 and an average of 3.45 per patient. The consultant gave recommendations regarding GP diagnosis (15.6%), GP treatment (52.8%), quality assurance (20.8%), referral (75.2%), institutions (2.9%), certificates (8.1%), work (10.4%), and social support (43.3%). Recommendations were preferably given in cases in which this particular treatment option had not been tried before. The most frequent recommendations were to attend a relaxation course (patients who have already been treated this way: 23.9%, new treatment: 48.3%, χ^2^ = 13.2; *P* < .001,), start leisure activities (already: 19.4%, new: 45.0%, χ^2^ = 8.5; *P* < .01), attend specialist psychotherapy (already: 29.1%, new: 61.3%, χ^2^ = 26.1; *P* < .001), begin sport activities (already: 25.0%, new: 22.1%, χ^2^ = 0.2; *P* = .69), initiate medication (already: 13.9%, new: 31.7%, χ^2^ = 13.8; *P* < .001), give patient education/bibliotherapy (already: 10.9%, new: 18.8%, χ^2^ = 1.7; *P* = .19), make application for inpatient rehabilitation (already: 7.9%, new: 20.6%, χ^2^ = 9.2; *P* < .01), reduce medication (already: 4.2%, new: 12.4%, χ^2^ = 2.7; *P* = .097), objectively monitor clinical course (already; 4.5%, new: 12.2%, χ^2^ = 2.2; *P* = .14), and intensified somatic diagnosis (already: 4.7%, new: 32.0%, χ^2^ = 37.7; *P* < .001).

For 89% of the patients, GPs accepted recommendations as reasonable and worth trying. In the remaining cases, they saw problems with the feasibility, necessity, or efficacy of the recommendations. We observed a rejection rate >3% for leisure activities, relaxation courses, start of a new medication, and patient education/bibliotherapy. There were no differences between the study groups.

### Changes in Treatment After Counseling

**Figure 1.**
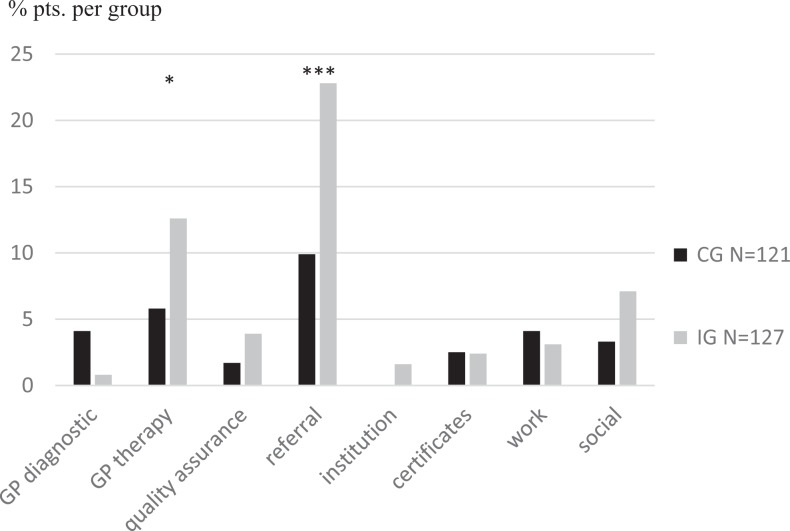
Percentage of patients per group with at least 1 implemented activity. The control group (CG) is shown in black, and the intervention group (IG) is shown in gray (*P < .05; **P < .001).

After 6 months, physicians and patients reported a higher rate of newly implemented actions in the IG than the CG (Figure 1). The differences in rates suggest a positive effect of the recommendations with respect to new treatments, initiated by GPs (CG: 5.8%, IG: 12.6%, χ^2^ = 3.4; *P* < .05) or referrals (CG: 9.9%, IG: 22.8%, χ^2^ = 7.5; *P* < .01). Of interest is that diagnostic measures were less frequent in the follow-up in the IG, as the case workup of the consultant may have clarified some diagnostic problems. The rates of newly implemented actions in the IG are considerably lower than in the CG.

We found differences between CG and IG for referrals (CG: 0.8%, IG: 5.5%, χ^2^ = 4.4; *P* < .05), sport (CG: 1.7%, IG: 7.1%, χ^2^ = 4.3; *P* < .05), relaxation (CG: 2.5%, IG: 7.9%, χ^2^ = 3.6; *P* < .05), leisure activities (CG: 2.5%, IG: 7.1%, χ^2^ = 2.9; *P* < .05), specialist psychotherapy (CG: 7.4%, IG: 14.2%, χ^2^ = 2.9; *P* < .05), and application for inpatient rehabilitation (CG: 0.0%, IG: 3.1%, χ^2^ = 3.9; *P* = .07).

### Effects of New Therapeutic Actions

When asked whether the newly started treatments had any effect in the IG, 47% of physicians and 51% of patients saw positive effects, and 5% of physicians and 5% of patients saw negative effects of the new interventions. In the CG, 63% of physicians and 56% of patients reported positive effects of the self-initiated interventions and 1% and 2%, respectively, observed negative effects.

Repeated measures ANOVAs showed improvements over time for IG and CG patients alike, but no significant interactions in symptom status (SCL-90-global severity index; time: *F* = 6.3, *P* = .013; interaction: *F* = 1.1, *P* = .29), global well-being (WHO-5; time: *F* = 26.8, *P* < .001; interaction: *F* = 3.3, *P* = .07), and participation (IMET; time: *F* = 14.21, *P* < .001; interaction: *F* = 1.06, *P* = .30).

## Discussion

The TOPPP checklist^3^ is a compilation of almost all conceivable interventions in the treatment of persisting psychological problems. The data suggest, firstly, that GPs use a broad range of diagnostic and therapeutic approaches, from medical treatments, to psychotherapy, to social support,^3,29^ and secondly, that the intensity and comprehensiveness of treatment is high.

Consultant recommendations are an indicator of unused options. Most often, rather unspecific measures were recommended, like encouraging leisure activities or stress management. Recommendations for new treatments were given as repetition of what had already been tried before, but preferably in cases without earlier respective interventions. For example, for 1 in 4 of those who had already been in psychotherapy, a retry was recommended, but even more so in 2 of 3 patients who had not been in psychotherapy before. In general, the consultant did not suggest any novel approaches but was proactive in trying to exploit any existing treatment possibilities.

The GPs appreciated such help, which may indicate their awareness of the difficulty in achieving remission for their patients. The fourth conclusion is that GPs are open to good advice from specialists and see most of the recommendations as useful.^30,31^ Accordingly, there were more new therapeutic actions initiated in the IG than in the CG, although there were also new actions in the CG. The fifth conclusion is that a consultant can stimulate new therapeutic actions. In about half of the patients, the GPs and the patients alike saw these as helpful, but about 5% saw them as negative. In the CG, the positive ratings on new therapeutic or diagnostic actions were higher and the negative ratings lower than in the IG, suggesting that actions which were initiated by the GP themselves may be more targeted than those which were initiated from consultant advice. This may explain why in total, the rate of implemented new actions is considerably lower than the rate of recommendations, which the physicians initially thought to be a good idea.

The clinical course and outcome did not differ between the IG and the CG. There are other studies^32^ that report an improvement in symptom severity after implementation of special treatment regimens. Our data suggest that GPs are, by and large, correct in refraining from many additional diagnostic or therapeutic actions, as they do not necessarily make a difference. The results suggest that the effective treatment options in chronic disorders are limited. Lastly, we conclude that persisting medical problems are not necessary indicators for insufficient treatment but rather for treatment resistance.^33^

Participating physicians in our study work in a densely populated region with a well-equipped health-care system; other GPs may work differently in other health-care structures. Therefore, a limitation for our study is that we can only show what can in a well-equipped health-care system. Another limitation is that we only had 1 consultant and 1 supervisor, who only represent singular medical viewpoints. Having more consultants would not have solved this problem but increased the variance in different opinions. In any case, this does not hinder us from concluding whether a consultation leads to treatment changes.
